# Case Report: Familial Hypocalciuric Hypercalcaemia and Hashimoto's Thyroiditis

**DOI:** 10.3389/fsurg.2020.00030

**Published:** 2020-06-16

**Authors:** Shahd Mobarak, Munir Tarazi, Harry Spiers, Anjali Santhakumar, Bence Forgacs

**Affiliations:** ^1^Department of Transplant and Endocrine Surgery, Manchester Royal Infirmary, Manchester University NHS Foundation Trust, Manchester, United Kingdom; ^2^Department of Endocrinology, Macclesfield District General Hospital, East Cheshire NHS Trust, Macclesfield, United Kingdom

**Keywords:** familial hypocalciuric hypercalcaemia, primary hyperparathyroidism, Hashimoto's thyroiditis, follicular variant papillary thyroid carcinoma, case report

## Abstract

**Introduction:** Hypercalcaemia can be caused by many disorders. Primary hyperparathyroidism is the leading cause with parathyroidectomy being the definitive management. Familial hypocalciuric hypercalcaemia is a rarer cause in which resection of the parathyroid tissue does not result in normalized serum calcium.

**Case presentation:** We report the unusual case of a 53-year-old lady who presented with hypercalcaemia and elevated parathyroid hormone with a presumed diagnosis of primary hyperparathyroidism. She remained hypercalcaemic after parathyroidectomy and was later diagnosed with familial hypocalciuric hypercalcaemia. During the first operation, a lymph node was also removed, and the histopathology report suggested a metastasis of follicular variant papillary thyroid carcinoma (FVPTC). After multi-disciplinary team (MDT) discussion, the patient underwent a second exploration where total thyroidectomy and removal of the other parathyroid glands were performed. Hypercalcaemia completely resolved on surgical resection of the thyroid and parathyroid tissue, however histopathology revealed normal parathyroid glands and florid Hashimoto's thyroiditis. The initial diagnosis of FVPTC in the lymph node was revisited and the final histopathology report suggested an accessory thyroid nodule with florid Hashimoto's thyroiditis mimicking a lymph node.

**Conclusion:** Our case demonstrates the diagnostic dilemma in hypercalcaemia that may lead a patient to undergo unnecessary invasive procedures; the misdiagnosis of FVPTC after the first operation resulted in a second more extensive procedure. Patients with no clear surgical target and urine CCCR in the gray/non-diagnostic area should be routinely offered genetic testing despite negative family history.

## Introduction

Primary hyperparathyroidism (PHPT) is the leading cause of hypercalcaemia and its definitive management is parathyroidectomy. Familial hypocalciuric hypercalcaemia (FHH) is a rarer cause of hypercalcaemia caused by a mutation in the calcium-sensing receptor (CaSR) gene. FHH can be diagnosed using 24-h urinary calcium to creatinine clearance ratio and hypercalcaemia does not resolve on resection of parathyroid tissue. We report the case of a 53-year-old lady who presented with hypercalcaemia and presumed hyperparathyroidism with a subsequent diagnosis of FHH.

## Case Report


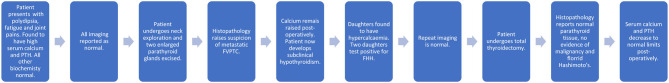


A 53-year-old lady presented in 2010 symptomatic with polydipsia, tiredness, and joint pains. At the time of presentation, no family history of hypercalcaemia was elicited. Examination of the neck was unremarkable with no goiter or lymphadenopathy. Biochemical investigations revealed normal renal function. Initial urine calcium creatinine ratio (CR) was 0.63 mmol/mmol (0.10–0.75 mmol/mmol). The CR was used as a surrogate marker for urine calcium creatinine clearance ratio (CCCR), which was calculated to be above 0.01 and did not meet the criteria for FHH. Parathyroid hormone (PTH) was elevated at 7.0 pmol/L (1.6–6.9pmol/L) and a corrected calcium was elevated at 3.03 mmol/L (2.2–2.6 mmol/L). Phosphate was 0.97 mmol/L (0.8–1.50 mmol/L). Vitamin D was 79.6 nmol/L (50–250 nmol/L). Thyroid function tests (TFTs) were normal (TSH 4.1 mu/L, T4 10.2 pmol/L). She had osteoporosis with a T score of −2.6 over the spine on a dual energy X-ray absorptiometry (DEXA) scan. No specific cause of the osteoporosis was identified on workup and this included normal full blood count, urea and electrolytes, liver function tests, vitamin D levels and TFTs; absence of long-term steroid use; no history of premature menopause and no history of long term physical inactivity. Renal ultrasound was normal.

**Figure 1:**
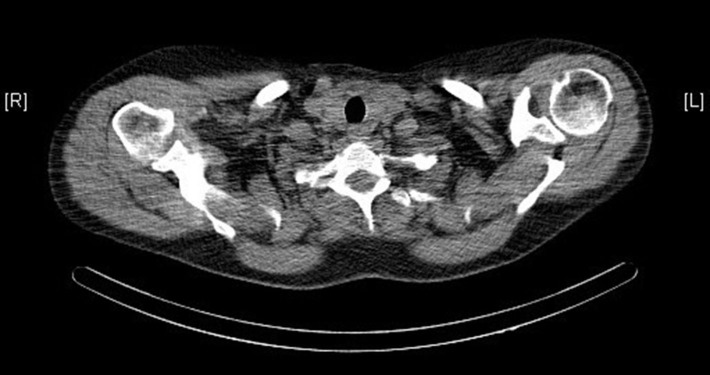
CT scan of the neck showing a normal thyroid gland.

Ultrasound, sestamibi scintigraphy and computed tomography (CT) scan of the neck did not demonstrate any parathyroid pathology (see Figure 1). On further investigation, a single photon emission computed tomography (SPECT) CT scan reported homogenous radiotracer uptake in the thyroid gland. The subtraction images showed subtle retention of activity in the lower medial aspect of the left thyroid gland, not convincing of parathyroid adenoma. Contrast CT of the neck noted no convincing radiological evidence of enlarged parathyroid adenoma and no lymphadenopathy in the neck.

Repeat biochemistry testing showed PTH elevated at 8.4 pmol/L, corrected calcium at 3.01 mmol/L, CR at 0.53 mmol/mmol and a vitamin D level of 35.8 nmol/L. She was started on vitamin D supplementation. She was initially managed medically, however due to persistent symptoms and evidence of osteoporosis, she was referred for a surgical opinion.

Despite the lack of radiological evidence, the decision for neck exploration and parathyroidectomy was made due to persistent symptoms exhibited by the patient along with the biochemistry supporting a diagnosis of PHPT and worsening osteoporosis (DEXA scan in 2018 showed a T score of −3.1 over the spine). At surgery, two slightly enlarged parathyroid glands were found in the left upper and lower positions, respectively, and excised. The right sided parathyroid glands appeared normal and so were left *in-situ* to avoid causing potentially avoidable lifelong hypocalcaemia.

**Figure 2:**
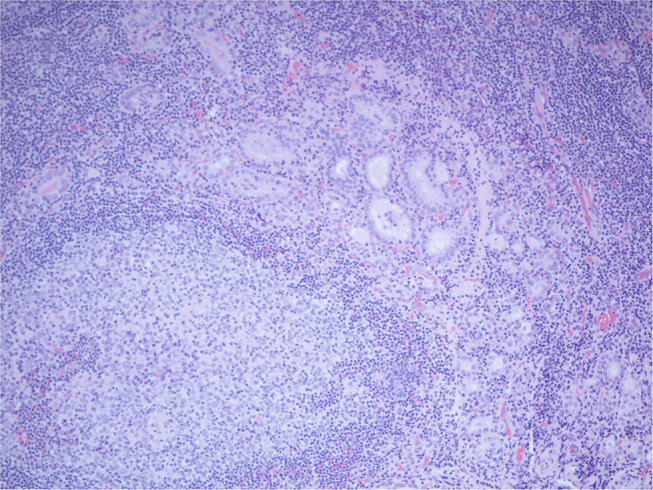
Histopathology image of the thyroid tissue suspicious for PTC.

Histopathology of the excised glands revealed only normal parathyroid tissue. However, an excised lymph node, that was presumed to be an enlarged parathyroid gland when excised, was found to contain numerous glandular/follicular structures which were identified as thyroid follicle inclusions in excess of normal numbers, alongside several papillary thyroid carcinoma (PTC) like nuclei (see Figure 2). This raised the suspicion of metastatic primary follicular variant PTC (FVPTC).

One-month post-operatively calcium and PTH levels remained raised at 2.91 mmol/L (2.2–2.6 mmol/L) and 8.0 pmol/L (1.6–6.9 pmol/L), respectively. Repeat TFTs were slightly deranged, TSH 6.6 mu/L, T4 10.6 pmol/L, suggesting subclinical hypothyroidism. Throughout the investigative process, the patient's calcitonin levels remained <1.0 ng/L (0–4.8 ng/L) thus a diagnosis of medullary thyroid carcinoma was excluded.

Patient informed us during her routine post-operative follow up that one of her previously fit and well daughters was admitted with hypercalcaemia. Her initial investigations showed a corrected calcium level of 3.12 mmol/L, PTH of 8.3 mmol/L, and CR of 1.52 mmol/mmol suggestive of a diagnosis of primary hyperparathyroidism. Her sestamibi scintigraphy was suspicious for a parathyroid adenoma. During her admission, her family links to our index patient were made and this led the endocrine team to revisit our patient's initial diagnosis. The differentials considered at the time were of familial hypocalciuric hypercalcaemia (FHH) as well as familial isolated hyperparathyroidism (FIHP). Her two other daughters and her grandchildren were contacted to have a serum corrected calcium level tested and all of them were revealed to have hypercalcaemia. Her son refused to have the necessary testing done. Most of them were asymptomatic apart from the daughter who presented acutely. None of their urine CCCR values were below the diagnostic cut off <0.01. Further history from the patient's general practitioner revealed that the patient's mother and brother had also suffered with hypercalcaemia but had never been investigated.

Subsequent genetic test was sent for two of her daughters which confirmed they were both heterozygous for pathogenetic CaSR variant consistent with a genetic diagnosis of FHH type 1. Multiple neuroendocrine neoplasia (MEN) was initially considered due to the strong familial link, however it was ruled out on further hormone testing including testing for metanephrines, IGF-1, fasting gut hormones, calcitonin and prolactin. Given its autosomal dominant mode of inheritance, our index patient was given a presumed diagnosis of FHH. The daughter with the suspicious parathyroid adenoma and confirmed FHH was referred to a tertiary center and remains under conservative medical management. Further imaging is planned to delineate the parathyroid adenoma identified on her sestamibi scan in order to aid the decision whether to operate on her.

Our patient underwent repeat SPECT CT scan which reported a slightly bulky right thyroid lobe with increased homogenous uptake compared to the left lobe, but no abnormal areas of increased uptake to suggest parathyroid adenoma. After a decision was made by the multi-disciplinary team (MDT), a total thyroidectomy was performed, confirming the scan findings of the right lobe being slightly bulkier than the left, with no nodules felt.

**Figure 3:**
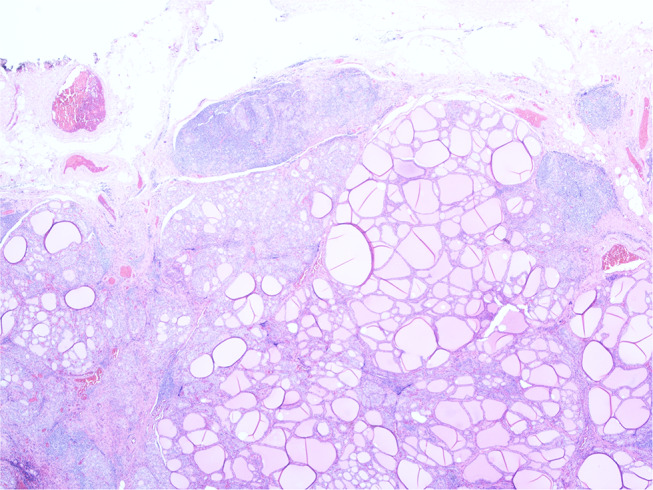
Histopathology image of the thyroid tissue showing florid Hashimoto's thyroiditis.

Histopathology revealed florid Hashimoto's thyroiditis throughout the thyroid gland, which also contained two morphologically normal parathyroid glands (see Figure 3). The previously excised lymph node biopsy was re-examined and found to be one of three possibilities: (1) benign thyroid inclusions in a lymph node; (2) an accessory thyroid nodule with florid Hashimoto's thyroiditis mimicking a lymph node, which was deemed most likely, or (3) metastatic papillary carcinoma from an occult micropapillary carcinoma despite extensive sampling.

Following total thyroidectomy, PTH levels dropped immediately [<0.6 pmol/L (1.6–6.9 pmol/L)] and the corrected calcium level began decreasing [2.62 mmol/L (2.2–2.6 mmol/L)]. The patient was started on liothyronine as well as calcium and vitamin D supplementation in light of both total thyroid- and parathyroidectomy. The histopathology specimen was reviewed at a tertiary cancer center which confirmed Hashimoto's thyroiditis and did not identify any malignant cells in the thyroid specimen, and so the patient was switched to levothyroxine for long term thyroxine replacement. Despite no definitive diagnosis, the patient remains well at follow-up, continuing her supplementation.

## Discussion

Our patient was doubly misdiagnosed: first with PHPT and subsequently with FVPTC. We will explore the reasons why these misdiagnoses occurred as well as recommend ways in which these can be avoided in the future.

The diagnosis of FVPTC was made on the presence of thyroid follicle inclusions in a lymph node on histopathology: in standard practice it would be negligent not to consider this a malignancy. The multi-disciplinary decision to proceed with a thyroidectomy was made in light of the information at the time. This misdiagnosis occurred primarily due to human error in investigating the microscopic features of the resected specimen.

The diagnostic difficulty between PHPT and FHH is often the main reason for misdiagnosis and consequently mismanagement in a patient. Factors proposed to help differentiate the two include (1): symptomatology (symptomatic in PHPT), bone density (decreased in PHPT), family history of hypercalcemia (positive in FHH), previous normocalcaemia (present in PHPT), and CCCR (<0.01 in FHH). Nonetheless, there remains significant overlap in these differentiating factors such as with this patient and her family which often makes it difficult to identify the exact etiology of the hypercalcaemia. FHH was initially not considered a likely cause for our patient as she was symptomatic with absent family history at presentation. Additionally, her CCCR was normal and she had evidence of osteoporosis. However, despite removal of the suspicious parathyroid tissue at the first neck exploration, the patient's serum calcium did not normalize, favoring a diagnosis of FHH later on in the clinical course. This is further supported by the subsequently determined strong family history of hypercalcaemia and confirmed by her daughters testing positive for CaSR gene.

CCCR is one test that can be done to differentiate between the FHH and PHPT, and the literature uses a cut-off point of <0.01 for FHH (sensitivity 0.796, specificity 0.876) and >0.02 for PHPT (2). However, some studies found approximately one-third of patients with confirmed PHPT have a CCCR in the range of 0.01–0.02 (3), thus, there remains a diagnostic dilemma for this group of patients. Specifically, for our patient, she did not meet the cut-off for FHH and thus a diagnosis of FHH was missed. Marx SJ (4) outlines in his review that, as PHPT is much more common, most patients with CCCR values near the 0.01 cut-off will have PHPT and not FHH. Consequently, a case can be made that such patients should undergo sequencing of their CaSR gene prior to parathyroid surgery.

One question our case raised is whether the combination of FHH and potential parathyroid adenoma in her daughter is a coincidental event or whether the adenoma is secondary to her CaSR mutation. In his review (4), Marx SJ postulates that given PHPT and FHH are found in 1 in 1,000 and 1 in 10,000 persons, respectively, their co-occurrence should be observed by chance in 1 in 10 million middle-aged women, making the combination a rare, but not improbable, event. Single or multiple parathyroid adenomas have recently been described in several family members in a setting of apparent FHH based on a mutation in the cytoplasmic tail of CaSR (5).

There have been few previous cases reported in the literature where patients with FHH were found to have a parathyroid adenoma, and in all cases, surgical resection resulted in the calcium levels improving but not normalizing (6–8). However, a recent study in Germany looked at 135 patients with hypercalcaemia who tested positive for FHH on genetic testing and found that four of them had features of PHPT (9). From the patients who underwent surgical resection, calcium levels decreased by 25% and normalized in 75% of patients. The authors concluded that this patient subset may benefit from parathyroidectomy, in keeping with our patient, where her serum calcium normalized following total parathyroidectomy.

Pathological examination of resected parathyroid glands in patients with FHH demonstrated glandular enlargement (10), most commonly mild parathyroid hyperplasia, but found no association with adenoma formation. One hypothesis is that the altered function of the CaSR gene results in fewer cell surface receptors, causing stimulation of excessive proliferation of the parathyroid cells. It is possible that the enlarged appearance of this patient's parathyroid glands at initial neck exploration could be attributed to this, although histology was not consistent with this.

Currently, genetic testing is the gold standard investigation. Identification of a heterozygous inactivating point mutation of the CaSR gene using polymerase chain reaction techniques can confirm a diagnosis of FHH. However, some patients may have novel mutations leading to atypical presentations (11) which contributes to the diagnostic dilemma of the cause of hypercalcaemia. In genetic variation, a familial form of CaSR-dependent hypercalcaemia has been described in association with other autoimmune disorders such as Hashimoto's hypothyroidism and celiac sprue in which autoantibodies directed against the sensor antagonize calcium recognition by the parathyroid glands and renal tubules (12, 13).

The presence of Hashimoto's thyroiditis and hypercalcaemia concomitantly is a case of incidence, however it raises the possibility of an etiological link between these two courses of pathophysiology. One retrospective study examined the link between thyroid pathology and PHPT by reviewing 65 patients who had undergone a neck exploration for hypercalcaemia (14). It found that 26 patients had associated thyroid pathology (40%), and three of those were nodules secondary to Hashimoto's thyroiditis. Only one of the three cases were detected pre-operatively. A laboratory study in rats using propylthiouracil to stimulate TSH, found that at 12 weeks, 95% of the rats showed hyperplasia of the parathyroid glands with a 30% mean increase in PTH (15).

In this case, our patient's serum calcium levels normalized following total thyroidectomy and parathyroidectomy, both of which occurred during the same operation. Therefore, removal of the four parathyroid glands was therapeutic in this case. However, the misdiagnosis of FHH pre-operatively could have perhaps been prevented had the patient underwent genetic testing for FHH, and this is something we have highlighted in this report.

Another factor is that the patient's phosphate on initial testing was normal. Although low phosphate is common in PHPT, it does not form part of the diagnostic criteria and hence PHPT was considered the differential diagnosis initially due to elevated PTH levels in absence of any other causes of the hypercalcaemia. However, we have considered that perhaps a normal phosphate along with a CCCR in the non-diagnostic criteria should encourage the clinician to look at an alternate diagnosis.

## Conclusion

Our case demonstrates the diagnostic dilemma in hypercalcaemia that may lead a patient to undergo unnecessary invasive procedures. Careful MDT involvement is crucial to help direct the best management for the patient and in this case for her larger family. Patients with no clear surgical target and urine CCCR in the gray/non-diagnostic area should be routinely offered genetic testing despite negative family history.

## Established Facts and Novel Insights

### Established Facts

- Primary hyperparathyroidism is the leading cause of hypercalcaemia.
- Definitive management of primary hyperparathyroidism is parathyroidectomy.
- Familial hypocalciuric hypercalcaemia is a rarer cause of primary hyperparathyroidism which cannot be treated effectively with parathyroidectomy.
- 24-h urinary calcium to creatinine clearance ratio is one test that can be done to differentiate between the two conditions.

### Novel Insights

- Familial hypocalciuric hypercalcaemia can occur concurrently with primary hyperparathyroidism, making it difficult to identify the cause of hypercalcaemia.
- Parathyroidectomy may normalize serum calcium in familial hypocalciuric hypercalcaemia.
- There may be an etiological link between Hashimoto's thyroiditis and hypercalcaemia.

## Data Availability Statement

The raw data supporting the conclusions of this article will be made available by the authors, without undue reservation, to any qualified researcher.

## Ethics Statement

Written informed consent was obtained from the individual(s) for the publication of any potentially identifiable images or data included in this article. The patient has given informed written consent for the case to be written and published in a medical journal.

## Author Contributions

BF, MT, and SM: conception and design. SM: literature search and obtaining the images. SM and MT: writing the article. All authors: critical revision and final approval of the article.

## Conflict of Interest

The authors declare that the research was conducted in the absence of any commercial or financial relationships that could be construed as a potential conflict of interest.
